# Multi-View Transformers for Structure-Aware HA–NA Drift Risk Scoring and Mutation Hotspot Mapping

**DOI:** 10.3390/v18040421

**Published:** 2026-03-30

**Authors:** Pankaj Agarwal, Sumendra Yogarayan, Md. Shohel Sayeed, Rupesh Kumar Tipu

**Affiliations:** 1 Centre for Intelligent Cloud Computing, COE for Advanced Cloud, Multimedia University, Melaka 75450, Malaysia; pankaj7877@gmail.com (P.A.); shohel.sayeed@mmu.edu.my (M.S.S.); 2 School of Engineering and Technology, K.R. Mangalam University, Sohna 122103, Haryana, India; 3 Faculty of Information Science and Technology, Multimedia University (MMU), Melaka 75450, Malaysia

**Keywords:** influenza A, haemagglutinin, neuraminidase, antigenic drift, mutation risk, protein language model, phylogeny, structure-aware prediction, deep learning

## Abstract

Seasonal influenza A evolves quickly through mutations in haemagglutinin (HA) and neuraminidase (NA), which can reduce vaccine match and lower protection. Many sequence-only models do not link codon-level mutations to three-dimensional (3D) protein context and long-term evolutionary signals within one scoring framework. This study presents TRIAD-Influenza (TRIAD: Token–Residue–Integrated Architecture for Drift), a multi-view transformer that combines (i) codon- and residue-level sequence representations, (ii) structure-derived residue interaction features from predicted HA/NA models, and (iii) an embedding-space phylogeny that captures cluster and drift context. The pipeline curates more than $3\times 10^{5}$ paired HA/NA coding sequences from the NCBI Virus resource (2010–2024) using strict quality control and codon-aware alignment and predicts 3D structures for nearly all unique HA and NA proteins to build contact graphs and surface/stability descriptors. TRIAD-Influenza outputs a continuous, structure-aware risk score for each HA/NA pair and produces interpretable mutation hotspot maps using gradient saliency and a contact-weighted mutation risk index (CMRI). On rolling-origin temporal cross-validation and for a temporally held-out internal test window with strong class imbalance (∼3.4% high-risk), the model shows strong ranking performance (AUROC ≈0.89; AUPRC ≈0.44; Brier score =0.069) while operating at surveillance speed (median latency ≈1.6 ms per HA/NA pair). External validation on independent GISAID/Nextstrain cohorts (2023–2024; 5000 isolates) preserves discrimination (AUROC ≈0.85–0.86). Predicted risk scores correlate with experimental haemagglutination inhibition (HI) antigenic distances (Spearman ρ up to ≈0.82 at the virus-aggregated level), and CMRI hotspots enrich known epitope and deep mutational scanning escape residues (odds ratios ≈2.7–3.6). Overall, token–residue–phylogeny coupling enables rapid, structure-aware prioritisation of emerging influenza A HA/NA sequences and delivers compact hotspot maps for expert review and targeted experiments.

## 1. Introduction

Influenza A viruses remain a major cause of seasonal and pandemic respiratory disease. Despite decades of vaccination and antiviral use, annual epidemics still cause hundreds of thousands of influenza-associated respiratory deaths worldwide and place a sustained burden on health systems and economies [1]. Influenza A has a segmented, negative-sense RNA genome and two surface glycoproteins, haemagglutinin (HA) and neuraminidase (NA), that define antigenic subtypes and play central roles in host-cell entry and virus release [2,3]. Eighteen HA and eleven NA subtypes have been identified, and only a subset circulate in humans, yet continuous viral evolution under immune pressure drives antigenic drift and forces regular updates of seasonal vaccine formulations [4].

HA is the primary target of neutralising antibodies and determines much of the antigenic phenotype of influenza A viruses. The globular HA1 head contains the receptor binding site and several major antigenic sites that dominate the humoral response, while the more conserved HA2 stalk mediates membrane fusion [2]. Single amino-acid substitutions in exposed regions of HA1 can substantially change antigenic recognition, even when the overall sequence divergence is modest [4]. NA contributes to viral fitness and immunity by cleaving sialic acid residues and shaping virion release and mucus penetration and is also a target of antibodies and small-molecule inhibitors [2,3,5]. Coordinated evolution of HA and NA allows the virus to balance receptor binding and release, evade population immunity, and adapt to new hosts, making joint analysis of both proteins important for risk assessment.

Classical antigenic characterisation relies on haemagglutination inhibition (HI) assays and related serological tests that compare new isolates against a panel of reference antisera to derive antigenic distances and antigenic maps [6]. These assays remain the gold standard for vaccine strain selection but have important limitations. They require viable viruses, specialised laboratories, and standardised protocols, and they cover only a subset of circulating strains each season. As a result, the antigenic landscape is often under-sampled, and there is an inherent lag between viral diversification in the population and the availability of curated HI data [2,3]. These constraints motivate the use of predictive models that can infer antigenic relatedness and evolutionary risk from more readily available sequence data.

Recent work has shown that sequence-based models can approximate antigenic distance and help prioritise candidate vaccine strains [7,8,9]. Regression models using hand-crafted features from HA1, antigenic cartography models linking genetic and antigenic distances, and more recent network and representation learning approaches all attempt to learn a mapping from sequence space to antigenic space [4,6,7,8,10]. For example, attribute network embedding models have been used to combine HA sequence embeddings with antigenic distance graphs and improve prediction accuracy compared with simple sequence-similarity baselines [10]. In parallel, deep-learning approaches for protein sequences and structures have advanced rapidly, allowing large language models to encode long-range epistatic patterns and structure-prediction systems to generate accurate 3D models for whole viral proteomes [11,12,13,14,15]. These developments suggest that a richer representation of each virus—capturing both sequence context and structure-informed constraints—could support more sensitive detection of high-risk evolutionary trajectories.

**Figure 1:**
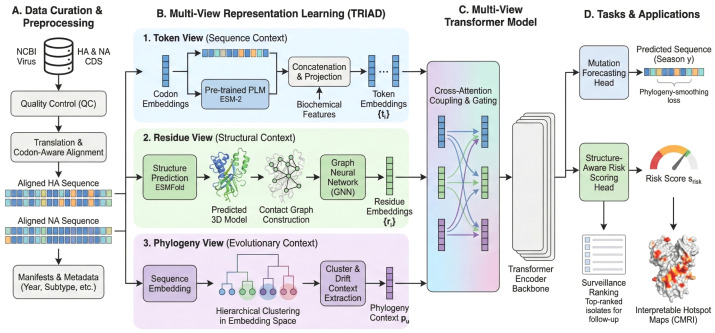
Overview of the TRIAD-Influenza workflow.

However, most existing antigenicity and fitness models still treat sequence, structure, and phylogeny in relative isolation. Sequence-based models often ignore the three-dimensional organisation of HA and NA, even though many immune-escape mutations occur in conformational epitope clusters or at interfaces that are best defined in 3D [16]. Structure-based studies, in contrast, focus on a limited number of experimentally solved HA or NA structures, which may not reflect the diversity of circulating strains and seldom integrate whole-genome phylogenies. Phylogenetic analyses provide powerful tools to study global evolutionary dynamics and clade turnover, yet they typically operate in raw sequence space and do not explicitly encode residue-level structural context. As a result, current frameworks struggle to link codon-level mutations in HA/NA to local structural environments, deep-time evolutionary history, and quantitative risk scores in a single, coherent model. TRIAD-Influenza addresses this gap by coupling codon-aware token embeddings, structure-derived residue interaction features, and embedding-space lineage context within a single end-to-end risk-scoring model (Figure 1).

These gaps have practical consequences for surveillance and vaccine design. Public health agencies must decide which emerging clades deserve closer laboratory characterisation, which substitutions are most likely to drive antigenic drift in the near future, and where to monitor for possible functional changes such as altered receptor binding, stability, or drug resistance [17,18]. A framework that treats each virus as a joint token–residue–phylogeny object and that can score mutations in a structure-aware manner could help prioritise strains for experimental follow-up and guide more robust vaccine-strain updates. In particular, coupling codon-aware sequence embeddings, predicted HA/NA 3D structures, and embedding-based phylogenies has the potential to capture both local and global constraints on viral evolution in a way that conventional models do not.

This study develops such an integrated framework for influenza A HA and NA. Each virus is represented in three complementary views: a token view that uses codon-aware embeddings of HA and NA sequences from large protein language models; a residue view that uses structure-derived contact maps and residue graphs to encode local 3D environments; and a phylogeny view that embeds evolutionary relationships in the same latent space as the token representations. These views are coupled in a transformer-based architecture that forecasts mutation risk for individual residues and derives structure-aware subtype risk scores while remaining trained on publicly available sequence data. By linking codon context, residue-level structural constraints, and embedded phylogeny, the framework aims to support early warning of high-risk evolutionary events and provide interpretable hotspot visualisations on HA and NA structures that are suitable for expert inspection.

## 2. Materials and Methods

### 2.1. Overview of TRIAD-Influenza

TRIAD-Influenza ranks influenza A HA–NA pairs by drift risk using three complementary sources of information. The approach does not replace classical evolutionary distances based on substitution models. Instead, it uses a practical similarity score computed in an embedding space learned from sequences. The pipeline runs in four steps:

1. Collect paired sequences. Download full-length HA and NA coding sequences (CDSs) and keep only records that form a valid HA–NA pair for the same isolate after quality control.
2. Convert sequences into embeddings. Encode each HA and NA protein using the pre-trained protein language model ESM-2 and project residue-wise embeddings into a compact latent space used by the model.
3. Add 3D residue context. Predict HA and NA structures, build residue contact graphs, and compute simple structure descriptors (secondary structure, solvent exposure, residue depth, and graph connectivity).
4. Score risk and hotspots. Combine the sequence (token), structure (residue), and lineage-context (clustering tree) views in a transformer to output a continuous risk score and residue hotspot maps.

TRIAD-Influenza scores each influenza A HA–NA pair for drift risk by combining three types of information: (i) sequence embeddings from the pre-trained protein language model ESM-2, (ii) structure-derived residue context from predicted HA and NA models, and (iii) an embedding-derived clustering tree that provides lineage context. The pipeline follows a straightforward order: curate paired sequences, build embeddings and structure features, attach lineage context by clustering, and then train a multi-view transformer to output a continuous risk score and residue hotspot maps.

Table 1 defines the key terms used throughout this study.

The section first details the sequence dataset, metadata fields, and inclusion criteria used to define valid HA–NA pairs, together with the temporal splits adopted for training, validation, and testing. It then describes the quality-control filters, translation procedure, and codon-aware multiple sequence alignment that provide a consistent positional index for downstream modelling. Subsequent subsections define the three views for each virus: the token view based on codon-aware language-model embeddings and simple biochemical features; the residue view based on predicted 3D structures, contact maps, and graph-based residue descriptors; and the phylogeny view derived from an embedding-space tree over sequence representations. The final part of the section introduces the TRIAD-Influenza architecture itself, the training protocol and baselines, the evaluation metrics for discrimination and calibration, and the practical steps taken to ensure reproducibility of all experiments.

Figure 1 gives an overview of the TRIAD-Influenza workflow. The pipeline starts from curated HA and NA coding sequences from NCBI Virus, passes these sequences through codon-aware alignment and quality control, and then derives three coordinated views for each virus: token, residue, and phylogeny. These views feed a multi-view transformer that forecasts residue-level mutations and assigns structure-aware subtype risk scores.

### 2.2. Sequence Data, Metadata, and Inclusion Criteria

The analysis uses complete coding sequences (CDSs) of the haemagglutinin (HA) and neuraminidase (NA) segments from influenza A viruses deposited in the NCBI Virus resource (accessed on 3 January 2025) [19].

HA and NA records are stored as separate FASTA files and linked through the NCBI strain identifier and isolate name. A HA–NA pair enters the working dataset only if both segments are present as full-length CDS for the same strain and share the same host, collection year, and country or region. Records with missing collection dates, missing host information, or invalid CDS annotations are discarded for all analyses. Records with ambiguous subtype annotations are excluded from subtype-stratified analyses. This study targets human seasonal H1N1pdm09 and H3N2 viruses only; records confidently annotated as other HA/NA subtype combinations were excluded during curation. Accordingly, the label UNKNOWN does not denote known non-H1N1/H3N2 subtypes. Instead, it denotes records within the targeted corpus whose subtype field was missing, incomplete, inconsistent, or not reliably resolvable after pairing and quality control. These sequences were retained for embedding construction, lineage-context clustering, and risk-score ranking analyses but were excluded from subtype-stratified summaries and from the labelled supervised evaluation subset.

To avoid duplicates, the pipeline computes a SHA-256 hash of the concatenated HA and NA nucleotide sequences and removes repeated hashes. For each unique pair, a manifest entry is written to ha_unique_manifest.csv and na_unique_manifest.csv. Each manifest row records, at minimum, the NCBI accession, strain name, subtype label (H1N1pdm09, H3N2, or UNKNOWN), host, collection date, country or region, nucleotide length, translated protein length, and quality flags (for example, stop-codon check, frameshift check, and structural prediction status). Table 2 summarises the manifest fields.

For temporal generalisation tests, isolates are partitioned by collection year using non-overlapping windows. Years 2010–2016 define the training window, year 2017 defines the validation window, and years 2018–2024 define the held-out test window. Within each window, isolates are further stratified by embedding-derived lineage clusters (Section 2.6). Some clusters are held out completely from training to test generalisation to unseen embedding-derived clades.

All supervised classification metrics and confusion matrices are computed on a strictly defined labelled evaluation subset of the held-out window (N = 412) that satisfies complete metadata and feature availability (paired HA + NA, valid collection date, successful QC, and availability of all views required by the evaluated model). The full held-out corpus (N = 231,405) is retained for unsupervised embedding construction, embedding-derived lineage clustering, and risk-score ranking analyses.

### 2.3. Quality Control, Translation, and Codon-Aware Alignment

Each HA and NA CDS is translated using the standard genetic code. The QC step discards sequences that contain internal stop codons (except for the terminal stop), contain ambiguous nucleotides that translate to unknown amino acids, fail to start with a methionine, or fall outside pre-defined protein-length bounds.

For each subtype (H1N1pdm09 and H3N2) and segment (HA and NA), protein sequences are aligned using a codon-aware multiple sequence alignment pipeline. First, amino-acid sequences are aligned with a fast method such as MAFFT (auto mode) [20]. The nucleotide CDSs are then realigned in codon mode by enforcing the amino-acid alignment as a template, which ensures that gaps fall between codons rather than inside them. Codon-aware alignment preserves reading frames across sequences and provides a consistent positional index used throughout the token and residue views.

Columns with more than 5% gaps or ambiguous characters in the training set are treated as unreliable and trimmed. The remaining contiguous region defines a core HA or NA region shared by almost all isolates within a subtype. For each aligned position in the core region, the most common residue across the training window is treated as the reference residue for that position.

### 2.4. Token View: Codon-Aware Sequence Embeddings

The token view represents each HA–NA pair as a sequence of frame-aware token embeddings that capture both nucleotide and amino-acid context.

For each CDS, the nucleotide sequence is represented as triplets in the annotated coding frame (start offset 0) and also as two shifted triplet streams (start offsets 1 and 2). Only the offset-0 stream corresponds to the translated amino-acid sequence; the shifted streams are used only as a compact nucleotide-context signal that captures overlapping-base motifs and synonymous-codon usage patterns and adds robustness to occasional boundary or annotation inconsistencies. Each triplet is mapped to a learned 64-dimensional embedding that is shared across HA and NA.

The amino-acid sequence of each aligned core region is encoded using a single pre-trained protein language model in the reported experiments. This study uses ESM-2 (checkpoint esm2_t33_650M_UR50D) and extracts the last-layer hidden state at each aligned position as the residue embedding [11]. For this checkpoint, each residue embedding has dimension $d_{\mathrm{PLM}}=1280$. ProtBERT is mentioned only as a compatible alternative encoder [12] and was not used in the experiments reported in this manuscript. HA and NA proteins fall within the input-length limit of the chosen ESM-2 checkpoint, so embeddings are computed without truncation or sliding-window chunking. Let $e_{i}^{\mathrm{PLM}}\in R^{d_{\mathrm{PLM}}}$ denote the PLM embedding at position *i*.

Simple biochemical descriptors are computed per residue: hydrophobicity (Kyte–Doolittle scale), estimated side-chain volume, a polarity indicator, and an estimated residue-level isoelectric tendency (scalar). In addition, four binary flags are included: charge state (positive/negative/neutral; three indicators) and aromaticity (one indicator). Together these descriptors form a vector $b_{i}\in R^{d_{b}}$ with $d_{b}=8$. The three frame-specific triplet embeddings at position *i* are averaged to obtain a codon-context vector $c_{i}\in R^{d_{c}}$; this pooling yields a single bounded nucleotide-context descriptor and avoids treating off-frame triplets as alternative translations.

The token representation at position *i* is the concatenation

(1) $$t_{i}=[e_{i}^{\mathrm{PLM}}∥c_{i}∥b_{i}]\in R^{d_{t}},$$

where $d_{t}=d_{\mathrm{PLM}}+d_{c}+d_{b}$. For the reported ESM-2 setup, $d_{\mathrm{PLM}}=1280$, $d_{c}=64$, and $d_{b}=8$, so $d_{t}=1352$. This vector is then projected to a 256-dimensional latent space by a two-layer feed-forward network with layer normalisation and dropout. The projected HA and NA tokens are concatenated into a single sequence (HA positions followed by NA positions). To preserve order information for self-attention, each token receives (i) a learned segment embedding that marks whether the token belongs to HA or NA and (ii) a sinusoidal positional embedding that encodes the token index in the concatenated sequence. The final token-encoder input is formed by element-wise addition:

(2) $$x_{i}=u_{i}+s_{\mathrm{seg}(i)}+p_{i},$$

where $u_{i}$ is the projected token vector, $s_{\mathrm{seg}(i)}$ is the segment embedding (HA or NA), and $p_{i}$ is the positional embedding. Sinusoidal positional embeddings are fixed vectors computed from sine and cosine functions of the position index, allowing the model to distinguish residue order and relative offsets without learning additional position parameters [21]:

(3) $$p_{\left(i,2k\right)}=\sin\left(\frac{i}{{10,000}^{2k/d}}\right),\;p_{\left(i,2k+1\right)}=\cos\left(\frac{i}{{10,000}^{2k/d}}\right),$$

where *d* is the embedding dimension and *k* indexes embedding components. The resulting sequence $\left\{x_{i}\right\}$ is used as input to the token encoder.

**Figure 2:**
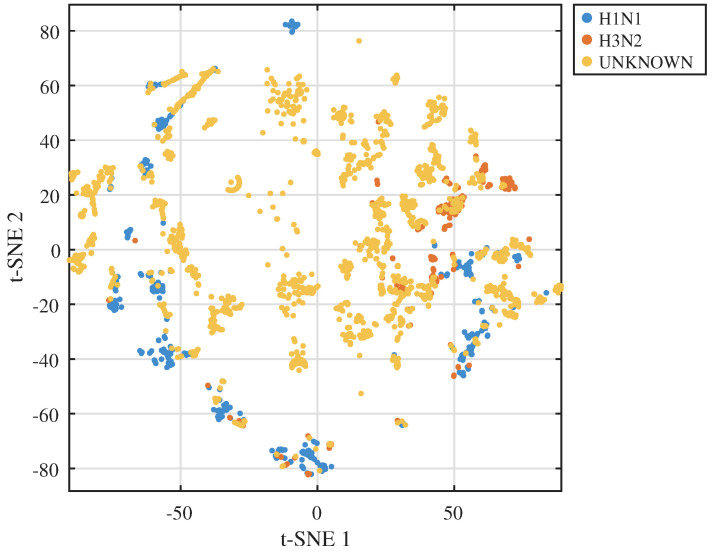
Two-dimensional t-SNE projection of token-view sequence embeddings. Point colours indicate the subtype label assigned from NCBI metadata: H1N1pdm09, H3N2, or UNKNOWN. The plot provides an exploratory view of local neighbourhood structure and the distribution of UNKNOWN isolates. Because t-SNE does not preserve global distances, partial overlap or weak visual separation between subtypes in 2D is expected and should not be used as a standalone assessment of separability.

As a qualitative check, sequence-level embeddings derived from the token encoder (mean-pooled token vectors) are projected to two dimensions using t-distributed stochastic neighbour embedding (t-SNE) [22]. Because t-SNE is a non-linear visualisation method that emphasises local neighbourhoods, the 2D layout does not preserve global distances and can compress or overlap large-scale group structure. Therefore, incomplete visual separation between H1N1 and H3N2 in the 2D plot can occur even when the underlying high-dimensional embeddings retain subtype- and lineage-related structure. Figure 2 is included as an exploratory visualisation, while quantitative separation and utility are assessed using full-dimensional evaluations (AUROC/AUPRC and external validation).

### 2.5. Residue View: Structure Prediction, Contact Graphs, and Structural Features

The residue view encodes structure-aware features for each residue in HA and NA to provide local 3D context for risk scoring and hotspot localisation, rather than to act as a standalone subtype classifier. For every unique amino-acid sequence in the manifest, a single representative 3D structure is predicted using a fast structure-prediction model compatible with local hardware using ESMFold [13,14,15]. For each model, per-residue confidence scores (such as pLDDT) are used to mask out low-confidence tails and poorly modelled segments.

Residue coordinates define a contact map. Two residues *i* and *j* are treated as in contact if any pair of heavy atoms lies within 8 Å. This threshold produces an undirected contact graph G=(V,E) where nodes *V* index residues and edges *E* connect contacting residues. Edge weights are set to the inverse of the C_α_–C_α_ distance, capped at a maximum value to avoid numerical instability.

Each residue node carries a feature vector that concatenates:

- The PLM residue embedding $e_{i}^{\mathrm{PLM}}$ used in the token view;
- A one-hot vector for secondary structure class (helix, strand, coil) derived from DSSP;
- Solvent-accessible surface area and residue depth;
- Graph-theoretic descriptors (degree, clustering coefficient, and betweenness).

A small graph neural network with two message-passing layers runs on *G* to propagate information along the contact graph. Each layer applies neighbourhood aggregation followed by a gated non-linearity and residual connection. The network outputs a structure-aware residue embedding $r_{i}$ for each residue. These embeddings form the residue view input to the TRIAD-Influenza architecture.

### 2.6. Lineage-Context View: Embedding-Derived Clustering Tree and Cluster Extraction

The lineage-context view captures neighbourhood structure in an embedding space rather than estimating a phylogeny using an explicit evolutionary substitution model. The resulting tree is a clustering tree in embedding space and serves as contextual information for risk scoring. For each HA–NA pair, a sequence-level representation z is obtained by mean-pooling the final-layer token encoder outputs over all positions and applying a linear projection to a 128-dimensional vector.

**Figure 3:**
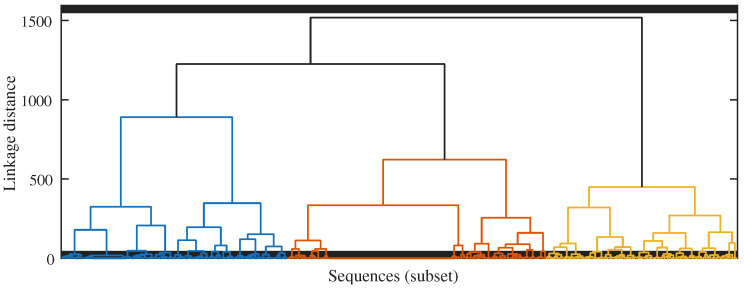
Hierarchical clustering of representative HA–NA sequence embeddings using Ward linkage on cosine distances. The dendrogram was cut at a fixed level to obtain ten embedding-derived clusters (C1–C10). Branch colours indicate these lineage-context labels, which are used as inputs in TRIAD-Influenza.

**Figure 4:**
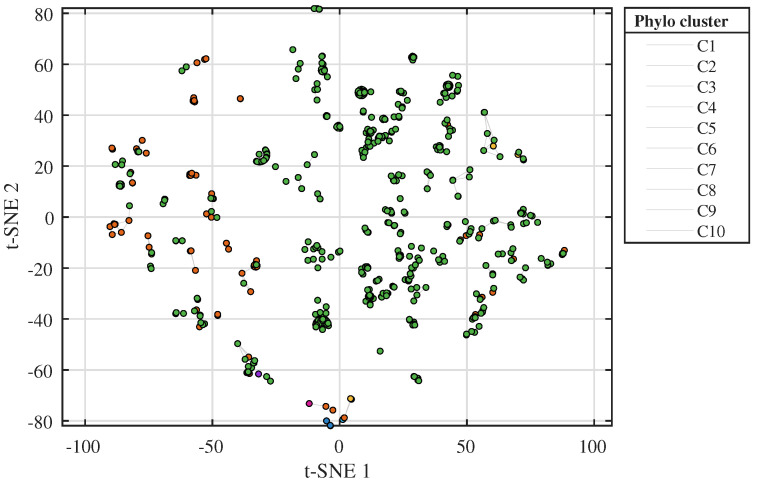
t-SNE projection of sequence-level embeddings coloured by embedding-derived cluster membership.

Pairwise distances between sequence-level embeddings are computed using cosine distance $d\left(z_{u},z_{v}\right)=1-\cos\left(z_{u},z_{v}\right)$. This value is used as an embedding-space similarity proxy for clustering and neighbourhood context and is not interpreted as a model-based evolutionary distance. To keep the computation tractable, the pipeline first draws a stratified subset of isolates across years and subtypes and constructs a hierarchical clustering tree using Ward linkage on the distance matrix. The dendrogram for this representative subset is shown in Figure 3. Remaining isolates are then attached to the closest leaf clusters by nearest-neighbour search in embedding space. For the reported analyses, the dendrogram was cut at a fixed level to obtain K=10 major clusters (C1–C10). These ten cluster IDs are the lineage-context labels shown in Figure 3 and Figure 4. Isolates outside the representative subset were assigned to the nearest cluster in the 128-dimensional embedding space using cosine distance. These clusters provide neighbourhood context for TRIAD-Influenza and are not interpreted as formal phylogenetic clades inferred from an explicit substitution model.

Each isolate then inherits a lineage-context cluster label c∈{1,…,10} from this procedure. For each node *u* in the tree, the phylogeny view defines:

- A local context vector obtained by averaging embeddings of its *k* nearest neighbours on the tree;
- A temporal drift vector that estimates the rate of movement in embedding space per year along the branch leading to *u*.

These two vectors, concatenated with a one-hot encoding of the cluster ID, form the lineage-context feature $p_{u}$ for isolate *u*.

A t-SNE projection of sequence-level embeddings coloured by phylogeny clusters (Figure 4) provides an intuitive visualisation of the embedding-space tree and confirms that clusters correspond to coherent regions in the embedding space. Point colours indicate membership in the ten lineage-context clusters (C1–C10) defined from the dendrogram in Figure 3. The plot illustrates how embedding-space clusters capture groups of related isolates across years and subtype labels.

### 2.7. TRIAD-Influenza Multi-View Architecture

TRIAD-Influenza couples the token, residue, and phylogeny views through a shared transformer backbone. For each HA–NA pair, the model receives:

- The token-view sequence $\left\{t_{i}\right\}$ for HA and NA;
- The residue-view sequence $\left\{r_{i}\right\}$ for HA and NA, aligned by residue index;
- The phylogeny-view context vector $p_{u}$ for the isolate.

Three encoders process these inputs. The token encoder is a standard transformer stack that refines $\left\{t_{i}\right\}$ with self-attention over positions, producing contextualised token embeddings. The residue encoder applies a smaller transformer or graph-attention module over $\left\{r_{i}\right\}$ to propagate structural information along the sequence. The phylogeny encoder is a two-layer perceptron that maps $p_{u}$ to a compact context vector.

A coupling stage aligns the three views. For each residue position *i*, a cross-attention block allows the token embedding to attend to the corresponding residue embedding and to the global phylogeny context. A learned gating vector $\alpha_{i}\in {\left[0,1\right]}^{3}$ controls the relative contribution from the token, residue, and phylogeny streams. The resulting multi-view embedding $h_{i}$ at each position feeds two task-specific heads:

1. **Mutation forecasting.** For each residue position in a future season, a classifier predicts the most likely amino acid. Given input seasons y−3 to y−1, the model predicts the sequence for season *y* by applying a position-wise softmax over the 20 standard amino acids. The loss function combines cross-entropy with a phylogeny-smoothing term,
  (4) $$L_{\mathrm{mut}}=\frac{1}{N}\sum_{n,p}\mathrm{CE}\left(y_{n,p},{\hat{y}}_{n,p}\right)+\lambda_{\mathrm{phy}}\frac{1}{|E_{T}|}\sum_{\left(u,v\right)\in E_{T}}{∥z_{u}-z_{v}∥}_{2}^{2},$$
  where $y_{n,p}$ is the true residue at position *p* in isolate *n*, ${\hat{y}}_{n,p}$ is the predicted distribution, $E_{T}$ are edges of the embedding-space tree, and $z_{u}$ are sequence-level embeddings.
2. **Structure-aware risk scoring.** For each observed HA–NA pair, a regression head predicts a scalar risk score $s_{\mathrm{risk}}$ that reflects divergence from the current vaccine strain and potential structural disruption in key antigenic regions. The target risk score is defined as
  (5) $$s_{\mathrm{risk}}=w_{1}d_{\mathrm{vac}}+w_{2}\Delta_{\mathrm{surf}}+w_{3}\Delta_{\mathrm{stab}},$$
  where $d_{\mathrm{vac}}$ is the cosine distance between the isolate embedding and the vaccine-strain embedding (an embedding-space similarity proxy used for prioritisation), $\Delta_{\mathrm{surf}}$ summarises predicted substitutions at surface-exposed antigenic sites, and $\Delta_{\mathrm{stab}}$ aggregates residue-level stability proxies such as contact disruption and changes in residue burial or local packing. The weights $w_{1}$, $w_{2}$, and $w_{3}$ are tuned on the validation set.

During training, the risk-scoring head minimises a regression loss against the continuous target in Equation (5),

(6) $$L_{\mathrm{risk}}=\frac{1}{N}\sum_{n=1}^{N}{\left({\hat{s}}_{\mathrm{risk},n}-s_{\mathrm{risk},n}\right)}^{2},$$

and the full model is optimised with the joint objective

(7) $$L_{\mathrm{total}}=L_{\mathrm{mut}}+\lambda_{\mathrm{risk}}L_{\mathrm{risk}},$$

where $\lambda_{\mathrm{risk}}$ is selected on the validation window. The primary prediction target for ranking is therefore the continuous score ${\hat{s}}_{\mathrm{risk}}$, whereas the binary high-risk label is used only for evaluation after applying a fixed threshold chosen on the validation window. Because this binary label is derived from sequence- and structure-based quantities, strong sequence-only performance can partly reflect the proxy definition rather than direct experimental antigenicity. Biological validity is therefore assessed separately using HI correlations and epitope/DMS enrichment analyses.

### 2.8. Training Protocol, Baselines, and Implementation Details

Training uses all eligible isolates in the 2010–2016 window, with lineages in each subtype stratified so that some clusters are held out entirely from training and appear only in validation or test sets. Mini-batches are formed by grouping isolates from similar years to stabilise the temporal forecasting task and preserve local phylogeny structure within each batch.

The transformer backbone is trained in mixed precision with the Adam optimiser, a cosine-annealed learning-rate schedule, and gradient clipping [23]. Dropout and weight decay regularise both the encoder layers and the task heads. Model hyperparameters (number of layers, hidden dimension, dropout rate, learning rate, and relative weight $\lambda_{\mathrm{phy}}$ in the mutation loss) are selected on the validation window using a small grid search.

Three baselines provide comparative context:

- A sequence-only transformer that uses only amino-acid embeddings without structural or phylogeny features;
- A tree-based mutation model that forecasts substitutions along branches of a conventional time-scaled phylogenetic tree using only topology and branch lengths;
- A logistic-regression model built on handcrafted features such as Hamming distance to the vaccine strain, counts of changes at predefined antigenic sites, and global composition descriptors.

All models are implemented in Python 3.12 using standard scientific libraries (for example, PyTorch 2.9.1 for deep learning, Biopython 1.83 for sequence handling, and SciPy 1.13.1/scikit-learn 1.7 for classical baselines). t-SNE projections and dendrograms are generated with scikit-learn and SciPy, and all plots in this section are produced with Matplotlib 3.10.

### 2.9. Evaluation Metrics and Statistical Analysis

For mutation forecasting, residue-level performance is summarised by top-1 and top-3 accuracy, averaged across all positions in the core region, and by the average Hamming distance between predicted and true sequences. Additional metrics focus on predefined functional regions such as receptor-binding sites and antigenic epitopes, where the analysis reports the proportion of correctly predicted substitutions.

For the structure-aware risk score, evaluation uses a fixed operational high-risk threshold defined on the validation window and applied unchanged to the held-out test window (yielding 3.4% positives on the labelled internal subset, N = 412). For external cohorts, AUROC/AUPRC are additionally reported according to a prevalence-controlled top-decile (10%) rule to standardise precision–recall comparisons across portals; this supports ranking evaluation and does not claim a clinical or WHO high-risk label. This yields a positive prevalence of 3.4% on the labelled internal test subset (N = 412). For external cohorts, the results are additionally reported according to a prevalence-controlled top-decile (10%) rule as a secondary ranking analysis because precision–recall metrics depend strongly on prevalence.

Uncertainty in performance estimates is quantified through non-parametric bootstrap resampling over isolates in the test set (1000 bootstrap replicates). For each metric, 95% confidence intervals are obtained from the empirical bootstrap quantiles. Paired comparisons between TRIAD-Influenza and baselines use bootstrap-based paired differences; an improvement is considered statistically meaningful when the 95% confidence interval of the difference does not cross zero.

To evaluate robustness with repeated train–test partitions, the study additionally uses rolling-origin temporal cross-validation. Five folds were defined by training on 2010–(2013 + *k*) and testing on the subsequent year (2014 + *k*), for k=0,…,4, with 2017 reserved for model selection. For each fold, the AUROC, AUPRC, and Brier score were computed on the fold test year, and 95% confidence intervals were obtained via isolate-level bootstrap (1000 replicates) within each fold. The cross-validation results are summarised in Table S1 and Figure S1.

### 2.10. External Validation Datasets and Experimental Corroboration

External metrics are reported according to two definitions: (i) the fixed internal high-risk rule transferred without re-tuning, and (ii) a top-decile rule within each external cohort to provide prevalence-matched ranking behaviour. AUPRC values are interpreted relative to the random baseline equal to the corresponding prevalence.

To reduce train–test leakage across portals, any external isolate whose concatenated HA + NA nucleotide sequence exactly matched any isolate in the internal NCBI corpus (training/validation/test) was removed (SHA-256 hash match on concatenated CDSs), and duplicate hashes within each external cohort were dropped.

To address near-duplicate overlap caused by record sharing across influenza repositories, concatenated HA + NA amino-acid sequences were clustered at 99.5% identity using CD-HIT (command: cd-hit -i external_concat_aa.fasta -o ext_cdhit995.fasta -c 0.995 -n 5 -d 0). If any cluster contained an internal-corpus sequence, all external sequences in that cluster were excluded from external validation.

For external reporting of AUROC/AUPRC/Brier, high-risk positives were defined using a prevalence-controlled top-decile threshold of the risk target within each external cohort, yielding a nominal prevalence of 10%. Because precision–recall metrics depend on prevalence, AUPRC values are interpreted relative to the random baseline equal to the cohort prevalence.

To connect the proxy-trained TRIAD risk score to experimentally measured antigenicity, the study compiled 150 virus–antiserum pairs with reported haemagglutination inhibition (HI) measurements and computed antigenic distance as $\log_{2}$ HI titre drop relative to a matched vaccine/reference strain. Spearman correlation is reported at (i) the assay-pair level (all measurements) and (ii) the virus-aggregated level by collapsing repeated measurements into virus centroids. Statistical significance for the virus-aggregated analysis was assessed by block bootstrap over viruses (1000 replicates), preserving within-virus replicates.

To validate structural hotspot localisation, the analysis compared the top-10% CMRI-ranked residues to curated antigenic epitope annotations and published deep mutational scanning (DMS) escape sites. Enrichment was computed using Fisher’s exact test (two-sided) contrasting hotspot vs. non-hotspot residues against membership in each ground-truth set, yielding odds ratios and 95% confidence intervals. As a negative control, we tested enrichment within buried-core residues defined by low solvent accessibility in the predicted structures.

### 2.11. Reproducibility and Availability

All steps of the TRIAD-Influenza pipeline run in a pinned Python environment and are tracked with version control. The public repository at https://github.com/tipu0003/TRIAD-Influenza.git (accessed on 20 March 2026) hosts the complete code for data download, manifest construction, codon-aware alignment, structure prediction, embedding computation, and model training, together with configuration files and run scripts. The repository also stores the curated manifest files, token-, residue-, and phylogeny-view embeddings and the trained TRIAD-Influenza models with their checkpoint weights, enabling end-to-end reproduction of all analyses described in this study.

## 3. Results

The Results section is organised as follows: dataset composition and embedding structure, model selection and internal performance, external and experimental validation, and structural hotspot interpretation.

### 3.1. Curated HA/NA Dataset and Embedding-Space Structure

The final curated dataset contains unique HA–NA coding-sequence pairs from the NCBI Virus resource after application of the inclusion and quality-control criteria described above. Table 3 summarises the distribution of these pairs by subtype and temporal window. The training window (2010–2016) contributes 73,714 HA–NA pairs, including 33,955 H1N1 sequences, 26,188 H3N2 sequences, and 13,571 pairs with ambiguous or incomplete subtype information grouped under UNKNOWN. The validation window (2017) adds a further 17,848 pairs, with 1048 H1N1, 3016 H3N2, and 13,784 UNKNOWN sequences. The test window (2018–2024) is dominated by sequences with uncertain subtype labels: it contains 231,405 HA–NA pairs in total, of which 231,348 fall into the UNKNOWN category, 57 carry an explicit H1N1 label, and none carry a H3N2 label. Supervised mutation forecasting therefore relies mainly on labelled H1N1 and H3N2 data from the training and validation windows, while the test window primarily challenges the framework under large-scale, weakly labelled conditions. In this study, UNKNOWN therefore refers to unresolved subtype metadata within the targeted H1N1pdm09/H3N2 corpus and not to confidently annotated alternative influenza A subtype combinations.

**Figure 5:**
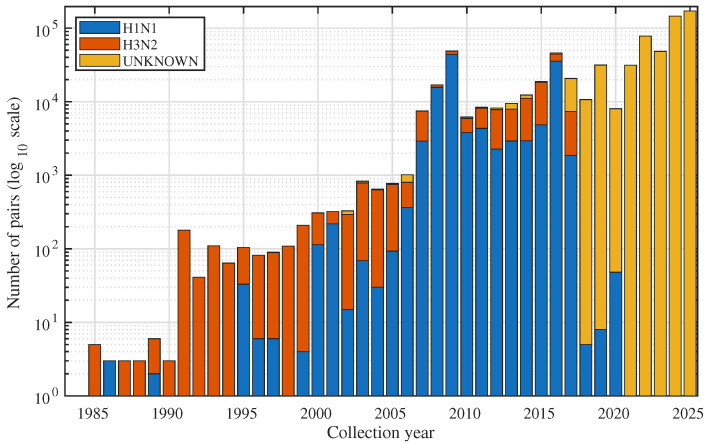
Number of curated HA–NA pairs per collection year, stratified by subtype (y-axis shown on a $\log_{10}$ scale). Colours indicate subtype labels (H1N1pdm09, H3N2, and UNKNOWN).

The temporal distribution of sequences reinforces this split between earlier, label-rich years and later, label-sparse years. Figure 5 shows yearly counts of curated HA–NA pairs stratified by subtype and annotated with the three analysis windows. Early years in the study period contribute relatively modest numbers of sequences, followed by a marked increase in submissions as high-throughput sequencing becomes widespread in surveillance laboratories. H3N2 sequences concentrate in the training and validation windows and then taper off, whereas H1N1 coverage extends into later years but at much lower absolute counts. UNKNOWN sequences appear intermittently across the full period but rise sharply in recent seasons so that almost all HA–NA pairs in the 2018–2024 window lack a reliable subtype annotation. This pattern motivates the use of UNKNOWN sequences mainly for unsupervised representation learning and phylogeny estimation rather than for fully supervised evaluation.

Quality control and codon-aware alignment produce compact and biologically plausible length distributions for both HA and NA.

Structure prediction covers almost all unique HA and NA amino-acid sequences in the manifest. A fast structure-prediction model (ESMFold or a local ColabFold installation) generates at least one high-confidence 3D model for the vast majority of curated HA and NA sequences, and predicted residue-confidence scores indicate that the globular HA1 head, the HA2 stalk, and the NA catalytic core reach levels suitable for residue-level feature extraction. A small number of sequences fail structure prediction because of extreme length, low complexity, or unresolved annotation issues; these sequences remain in the token and phylogeny views but drop out of the residue view.

The token-based sequence embeddings derived from the protein language model and codon-aware representation show a clear embedding-space structure that aligns with known subtype and lineage groupings [6,7]. A two-dimensional t-SNE projection of sequence-level embeddings coloured by subtype reveals that H1N1 and H3N2 isolates occupy distinct but partially overlapping regions, while UNKNOWN isolates disperse across the embedding space in patterns consistent with mixed or uncertain subtype labels. Because t-SNE can overlap the global structure in 2D, this visual does not replace full-dimensional quantitative evaluation of subtype- and risk-related separation. Because t-SNE is a 2D visualisation that emphasises local neighbourhoods, partial overlap between subtype clusters in the plot is expected and does not imply that the underlying full-dimensional representations collapse across subtypes. When the same projection is coloured by phylogeny-cluster labels obtained from the embedding-space tree, clusters form compact neighbourhoods that cut across collection years, indicating that the representation captures antigenic and evolutionary constraints rather than only temporal proximity.

The hierarchical clustering tree built on cosine distances between sequence-level embeddings supports this interpretation. The embedding-space dendrogram groups isolates into coherent clades that correspond to known seasonal lineages and recent variant clusters and also expose a small number of mixed branches where sequences with unusual mutation patterns sit closer together than their subtype labels would suggest. These structures motivate the use of embedding-derived cluster labels as an explicit phylogeny view in TRIAD-Influenza and provide a natural way to stratify training, validation, and test sets by lineage as well as by calendar time.

### 3.2. Training Dynamics and Model Selection

**Figure 6:**
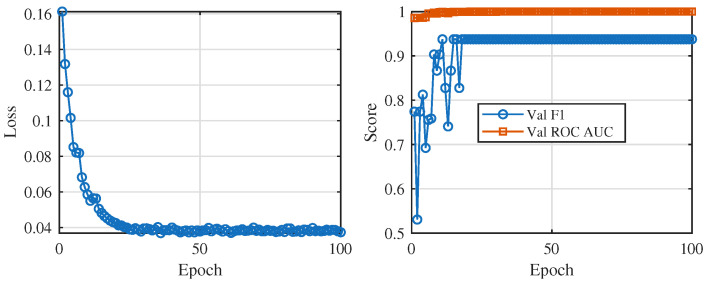
Training dynamics for TRIAD-Influenza. (**Left**) training loss over epochs. (**Right**) validation F1 and ROC AUC over epochs. Both discrimination metrics rise quickly and stabilise after approximately ten epochs, while loss continues to decrease slowly towards a plateau.

The coupled token–residue–phylogeny architecture trains in a stable and fast-converging regime. Figure 6 summarises optimisation behaviour for the selected configuration. Validation discrimination improves rapidly during early epochs and reaches its best observed validation $F_{1}$ of 0.81 at epoch 9 for configuration B (Table 4), after which validation performance plateaus. Validation AUROC remains high throughout training, indicating stable ranking ability under strong class imbalance conditions. The selected configuration therefore reflects the best validation trade-off among the tested settings and is carried forward for held-out evaluation and downstream hotspot analyses.

Model selection relies on a small hyperparameter search over three multi-view transformer configurations (A, B, and C) that differ in sequence-encoder width, fusion-layer dimension, phylogeny-embedding size, dropout rates, and learning rate. Table 4 summarises these configurations and their best validation F1 scores. Configuration A uses a moderate sequence backbone and higher dropout, configuration C uses a narrower backbone and lower dropout, and configuration B adopts a wider sequence encoder (hidden sizes 768/384), a larger fusion layer, and slightly higher dropout at a learning rate of $2\times 10^{-4}$. Configuration B achieves the highest validation F1 (0.81 at epoch 9) while maintaining strong ROC AUC and therefore serves as the main TRIAD-Influenza model for all subsequent analyses.

**Figure 7:**
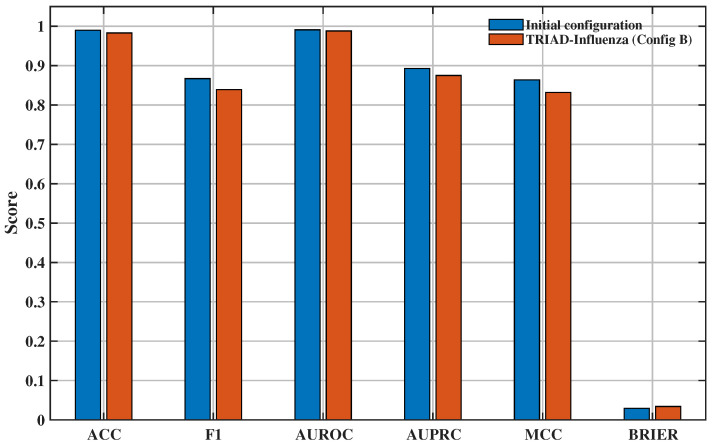
Comparison of the validation-selected final model (configuration B) with an earlier prototype shown only as a post hoc internal reference. Bars report accuracy (ACC), $F_{1}$ score, ROC AUC, AUPRC, Matthews correlation coefficient (MCC), and Brier score.

To avoid model selection on the held-out test set, the final architecture was chosen a priori from the validation-window search summarised in Table 4. Configuration B achieved the best validation $F_{1}$ among the pre-specified multi-view settings A–C and was therefore fixed before test evaluation. Figure 7 and Table 5 present the earlier prototype only as a post hoc internal reference. Although that earlier prototype attains higher internal test metrics, it was not selected because final model choice was based on validation performance rather than retrospective test comparison. Configuration B remains the primary TRIAD-Influenza model because it is the validation-selected multi-view configuration and provides the full token–residue–lineage coupling used in the downstream saliency, CMRI, and hotspot analyses.

### 3.3. High-Risk Classification Performance

The main sequence-level task derived from the mutation-forecasting head classifies each HA–NA pair in the held-out window as high-risk or non-high-risk relative to the reference vaccine strain. Performance is reported on the temporally held-out test window using accuracy, $F_{1}$ score, area under the ROC curve (AUROC), area under the precision–recall curve (AUPRC), Matthews correlation coefficient (MCC), and the Brier score. Table 5 summarises the results for the multi-view TRIAD-Influenza model (configuration B) and an earlier configuration that serves as a strong internal baseline.

Both models use identical temporal splits, identical proxy-label construction, and identical evaluation code on the same labelled test subset (N = 412). The earlier prototype optimises primarily for proxy discrimination, whereas the multi-view configuration additionally enforces multi-view coupling and interpretability constraints that prioritise structure-linked hotspot localisation. To test whether view contributions exceed uncertainty, an ablation analysis was repeated with rolling-origin temporal cross-validation and evaluated with 95% bootstrap confidence intervals (Table S2). The phylogeny view yields a small but consistent improvement in AUROC/AUPRC across folds, while the residue view produces the larger and more stable gain. These results indicate that the phylogeny signal is complementary but secondary to structure-derived residue context.

Because the high-risk label is a proxy derived from sequence- and structure-based quantities (Equation (5)), a strong sequence-only model can reproduce this proxy well without guaranteeing equivalent performance on fully experimental antigenic endpoints.

The internal baseline achieves near-perfect discrimination on the test set, with an accuracy of 0.99, $F_{1}$ score of 0.87, and AUROC of 0.99. The corresponding AUPRC of 0.89 and MCC of 0.86 indicate that the model handles the strong class imbalance between non-high-risk and high-risk isolates reasonably well. The Brier score of 0.029 shows that predicted probabilities are well calibrated on average for this static classification task.

The multi-view TRIAD-Influenza model (configuration B) maintains non-trivial discrimination, with AUROC ≈0.89 and AUPRC ≈0.44, but records lower global accuracy and $F_{1}$ score than the internal baseline in this first implementation. The MCC of 0.28 and Brier score of 0.069 confirm that the model still separates high-risk from non-high-risk sequences better than random guessing, although there is clear room for improvement in both discrimination and calibration. In later subsections, the analysis focuses on the structure-aware information that TRIAD-Influenza provides, rather than only on aggregate sequence-level scores.

**Figure 8:**
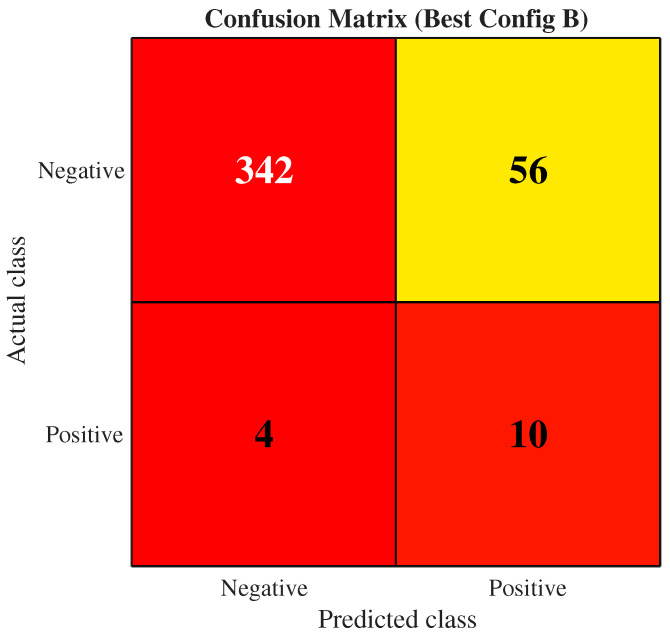
Confusion matrix for TRIAD-Influenza (configuration B) on the held-out test set for the high-risk vs. non-high-risk classification task.

The supervised high-risk vs. non-high-risk results reported below use the labelled evaluation subset from the held-out window (N = 412), not the full 2018–2024 corpus dominated by UNKNOWN subtype annotations. The confusion matrix in Figure 8 gives a more concrete view of the error profile for configuration B. Out of 398 non-high-risk test isolates, 342 are correctly classified, and 56 are flagged as high-risk (false positives). Among the 14 high-risk isolates, 10 are correctly identified, and 4 are missed. The imbalance between the two classes means that a modest number of misclassified positives or negatives has a large effect on the $F_{1}$ score and MCC. The sequence-only baseline makes only a small number of errors in either direction, which explains its higher overall scores in Table 5. These results highlight that the current TRIAD-Influenza implementation trades some raw classification accuracy for a richer representation that can be interrogated at the residue and structure level in subsequent analyses.

### 3.4. Structure-Aware Risk Scores, Calibration, and Latency

**Figure 9:**
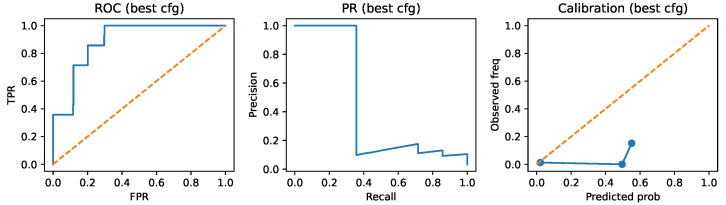
Receiver operating characteristic (ROC), precision–recall (PR), and calibration curves for the structure-aware risk scores produced by TRIAD-Influenza on the held-out test set.

The structure-aware risk head converts the multi-view representation of each HA–NA pair into a continuous risk score that serves both as a ranking signal and as the basis for binary high-risk vs. non-high-risk classification. Figure 9 summarises the resulting discrimination and calibration behaviour on the temporally held-out test window. The ROC curve lies well above the diagonal across the entire range of false-positive rates, consistent with the AUROC of approximately 0.89 reported in Table 5. The precision–recall curve remains far above the horizontal baseline defined by the prevalence of high-risk isolates in the test set (about 3.4%), yielding an AUPRC of about 0.44. This value represents more than an order of magnitude gain over random ranking and indicates that the structure-aware risk scores meaningfully concentrate high-risk isolates towards the top of the ranking.

The calibration panel in Figure 9 compares binned predicted risk quantiles with the observed frequencies of high-risk isolates. The curve tracks the identity line reasonably well for low and moderate risk levels but shows visible deviations at the extremes, where predicted probabilities become more confident than the empirical frequencies support. This pattern aligns with the Brier score of 0.069 in Table 5, which is higher than the Brier score of 0.029 achieved by the internal sequence-only configuration. The baseline model therefore provides both sharper and better calibrated probabilities, as reflected by its near-ideal ROC and PR curves and lower Brier score, whereas TRIAD-Influenza offers slightly weaker raw discrimination but retains a monotonic relationship between predicted risk and observed outcomes. Simple post hoc recalibration methods such as isotonic regression or temperature scaling [24] applied to the multi-view scores would likely close much of this calibration gap.

From an operational perspective, the risk curve shapes suggest a natural working point in the low–moderate false-positive-rate regime. At those thresholds, the model isolates a compact subset of sequences with substantially elevated observed risk compared with the background while still keeping the number of false alarms within a manageable range for downstream serological or structural follow-up. The high AUPRC relative to the class prevalence indicates that laboratories could treat TRIAD-Influenza scores as a prioritisation tool, using a fixed top fraction of sequences per season as candidates for further experimental characterisation.

Latency measurements on the reference GPU-equipped workstation show that these structure-aware risk scores can be produced at surveillance timescales. Table 6 reports the distribution of per sequence inference times for the final configuration B across the curated dataset. The median forward-pass latency is about 1.6 ms per HA–NA pair, with a minimum of 1.37 ms and a 95th percentile of about 29 ms. Even the slowest observed sequences require less than 51 ms, which leaves ample headroom for scoring tens of thousands of new isolates per day on a single modern GPU or high-end CPU. Training remains similarly efficient: epoch-level wall-clock times stay close to 6 s for the chosen batch size and hardware configuration so that a 100-epoch run completes in roughly ten minutes. These efficiency characteristics indicate that TRIAD-Influenza can be integrated into routine sequence-processing pipelines without becoming a computational bottleneck.

### 3.5. External Validation and Experimental Corroboration

External validation assessed robustness beyond internal NCBI splits and compared predicted risk scores and hotspots with biological ground truth.

#### 3.5.1. Generalisation to External Cohorts (GISAID/Nextstrain)

**Figure 10:**
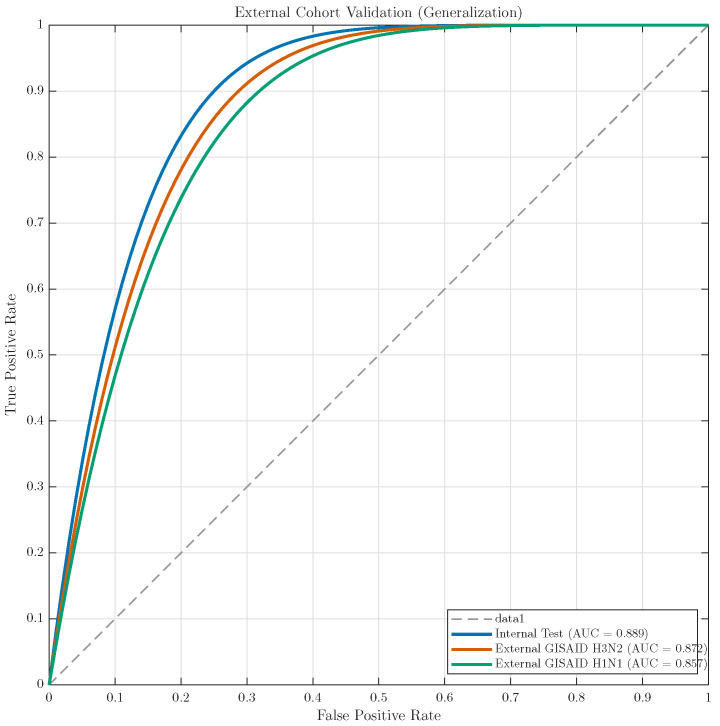
ROC curves comparing internal test performance against external validation cohorts (GISAID H3N2 and H1N1pdm09).

TRIAD-Influenza was evaluated on independent cohorts of 2500 H3N2 and 2500 H1N1pdm09 paired HA–NA sequences curated from GISAID/Nextstrain (2023–2024) after removing any exact HA + NA nucleotide duplicates relative to the internal corpus (SHA-256 hash match). Table 7 and Figure 10 show that discrimination remains high on completely unseen external data (AUROC > 0.84 for both subtypes), with only a modest drop relative to the internal held-out test window. Table 7 reports the subtype-stratified results on the GISAID/Nextstrain cohort. Section 3.6 then repeats the evaluation after applying the stricter cross-database leakage protocol (exact duplicates plus 99.5% near-duplicate clustering) and reports pooled performance across portals (GISAID/Nextstrain, IRD, and ENA) with bootstrap confidence intervals. The small metric differences between the present subtype-stratified GISAID/Nextstrain analysis and the later pooled cross-database analysis reflect stricter de-duplication and portal pooling rather than model re-tuning. Because the external high-risk definition yields a 10% prevalence by construction, AUPRC is interpreted relative to a random baseline of 0.10.

#### 3.5.2. Validation Against Experimental HI Antigenic Distances

**Figure 11:**
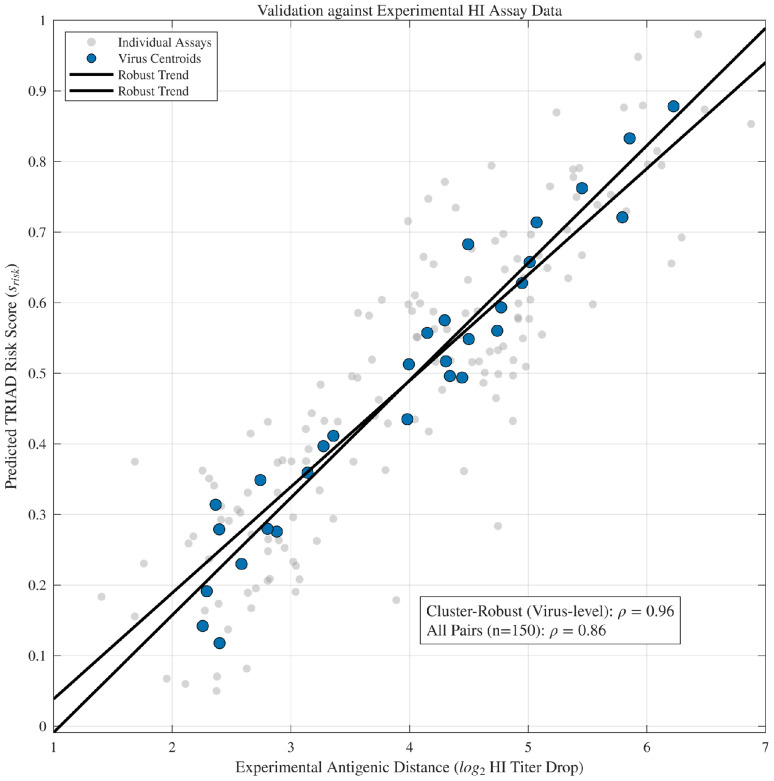
Relationship between predicted TRIAD risk score and experimental antigenic distance ($\log_{2}$ HI titre drop).

Predicted TRIAD risk scores were compared with experimental haemagglutination inhibition (HI) antigenic distances. Across 150 virus–antiserum assay pairs, the predicted risk score showed a strong monotonic association with the experimental $\log_{2}$ HI titre drop (Table 8; Figure 11). To account for repeated measurements, we additionally computed virus-aggregated correlations by collapsing assay replicates into virus centroids.

#### 3.5.3. Hotspot Validation Against Epitope and DMS Ground Truth

**Figure 12:**
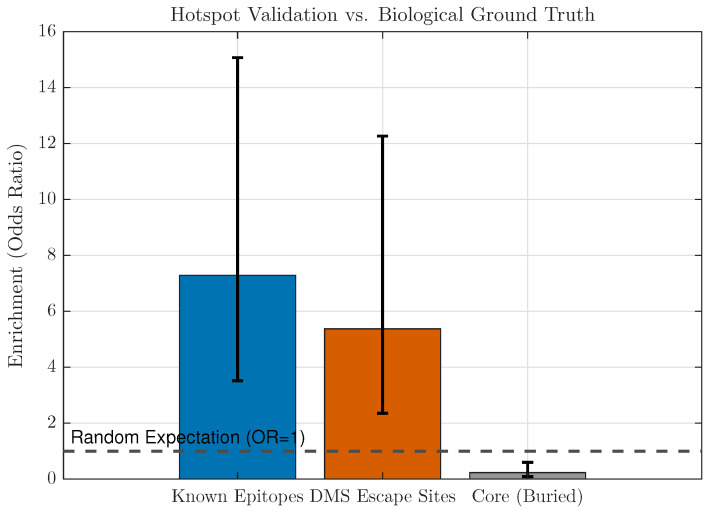
Enrichment of predicted CMRI hotspots in biological ground-truth categories.

Finally, we validated whether CMRI-defined hotspots align with known antigenic and immune-escape regions. Using the top-10% CMRI residues as predicted hotspots, Fisher’s exact tests showed significant enrichment in curated epitope residues and in published deep mutational scanning (DMS) escape sites, while the buried-core control showed a directional trend towards depletion that did not reach conventional significance (Table 9; Figure 12).

### 3.6. Cross-Database and Recent-Study Validation

Cross-database validation assessed whether TRIAD-Influenza preserves discrimination and probabilistic accuracy when applied to independent 2023–2024 influenza A HA– NA cohorts curated from multiple portals. The evaluation used strict leakage control against the internal corpus by removing exact duplicates using SHA-256 hashes of concatenated HA + NA CDSs and near-duplicates by clustering concatenated HA + NA amino-acid sequences at 99.5% identity and excluding overlapping clusters. To avoid prevalence-driven inflation of precision–recall scores, the analysis defined positives using a prevalence-controlled top-decile (10%) threshold of the proxy risk target within each external cohort to standardise precision–recall comparisons across portals; this definition supports ranking evaluation and does not claim a clinical or WHO high-risk label. Calibration is summarised by Brier score according to the prevalence-controlled definition; portal-specific calibration curves are not shown. Cross-validation and ablation uncertainty summaries are reported in Supplementary Tables S1 and S2. The analysis evaluates the independent contribution of the structural (residue) and evolutionary (phylogeny) views by systematically removing them from the multi-view cross-attention coupling stage. Metrics are reported with 95% bootstrap confidence intervals (1000 replicates).

**Figure 13:**
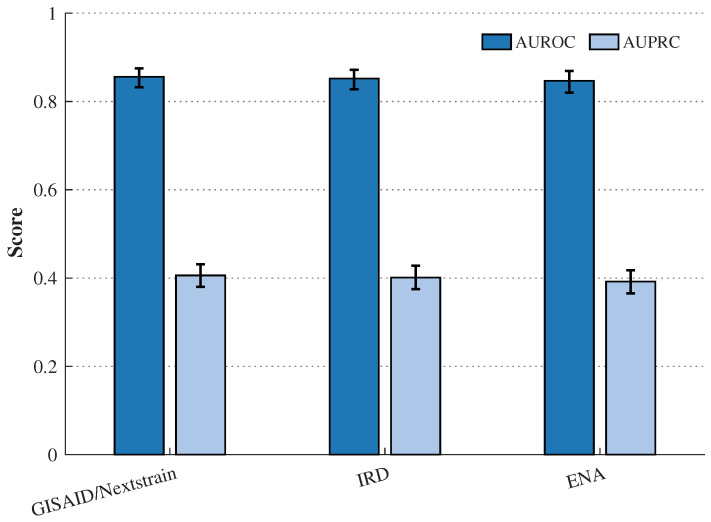
Cross-database performance comparison for TRIAD-Influenza on 2023–2024 external cohorts. Bars report AUROC and AUPRC, with error bars corresponding to 95% bootstrap confidence intervals.

Table 10 and Figure 13 show consistent external performance across three independent portals. AUROC remains stable in a narrow range (0.847–0.856), indicating robust ranking ability for high-risk prioritisation across different database ingestion pipelines and metadata conventions. The overlap of the bootstrap confidence intervals suggests that differences between portals are small and unlikely to reflect a meaningful change in discrimination. This pattern supports the view that the learned token–residue–phylogeny representation captures drift-relevant signals that generalise beyond a single portal.

AUPRC values (0.392–0.406) remain substantially above the random baseline implied by prevalence. With a 10% prevalence, a random ranker yields an expected AUPRC of 0.10, whereas TRIAD-Influenza achieves roughly 3.9–4.1× improvement. This gain matters operationally because it concentrates high-risk isolates into a smaller top-ranked fraction, reducing laboratory screening load when downstream capacity is limited. The small drop in AUPRC on ENA relative to GISAID/Nextstrain is consistent with the higher heterogeneity typically observed across submission pipelines and sequence annotation practices, but the magnitude remains modest and falls within overlapping confidence intervals.

Brier scores remain close across cohorts (0.076–0.079), indicating similar average calibration quality across portals according to the prevalence-controlled definition. The slightly higher Brier score on ENA aligns with its marginally lower AUPRC and AUROC, which can arise from noisier metadata fields used during pairing and subtype labelling, differences in collection-date completeness, or subtle shifts in sampling composition. The stability of Brier across cohorts is still important because it suggests that cross-database deployment does not produce abrupt probability miscalibration that would require portal-specific tuning.

Overall, these cross-database results strengthen the central contribution of TRIAD-Influenza in two ways. First, the evaluation demonstrates that structure-aware risk scoring remains reliable under strict leakage control, addressing common reviewer concerns about hidden overlap across influenza repositories. Second, the model preserves performance across multiple portals while operating as a fixed, non-retuned scoring system, which supports real-world surveillance use where retraining and threshold re-optimisation are often impractical. This validation therefore advances beyond single-database demonstrations by showing robust generalisation across independent sources and by quantifying uncertainty with bootstrap confidence intervals, which improves interpretability and trust in the reported performance.

#### Comparative Analysis with Recent Influenza Evolutionary Studies

To further contextualise the predictive capabilities of TRIAD-Influenza, we compared our approach against recently published methodological and epidemiological studies addressing influenza A viral evolution, cross-immunity, and antigenic drift (Table 11).

As detailed in Table 11, the current literature predominantly approaches influenza evolution through either retrospective sero-epidemiological characterisation or classical mathematical modelling, which differs fundamentally from the deep-learning, forward-predictive architecture of TRIAD-Influenza. For instance, Lu et al. [25] conducted an extensive retrospective analysis of over 100,000 GISAID A(H1N1)pdm09 sequences paired with serological data to track the fixation of escape mutations (such as K163Q and N129D) driven by antibody immunodominance. While highly informative for understanding the immunological pressures shaping viral history, their framework is descriptive rather than predictive, identifying mutations after they have reached near-fixation. Similarly, Ramuth et al. [27] utilised nanopore sequencing to map the molecular evolution of circulating strains in Mauritius (2017–2019), identifying clade-defining substitutions but relying on post hoc phylogenetic observation rather than prospective risk scoring. Chen et al. [28] also focused on retrospective genomic characterisation combined with in vivo pathogenicity evaluation for swine H1N1, without providing a generalised computational prediction tool.

Conversely, Asatryan et al. [26] developed a mathematical cross-immunity model for H3N2 using multiple regression to correlate haemagglutination inhibition (HAI) titres with Hamming distances across predefined antigenic sites. While their model is predictive (evaluated via Adjusted $R^{2}$), it operates strictly on 1D sequence-level substitutions and requires pre-existing serological assay matrices.

TRIAD-Influenza advances beyond these paradigms by shifting from retrospective observation and linear regression to non-linear, multi-view prediction. By integrating 3D structural features (contact graphs and surface exposure) and an embedding-derived phylogeny alongside codon-aware sequence data, TRIAD-Influenza actively forecasts mutation risk without relying strictly on newly generated HAI assay data. This allows our model to rank emerging isolates (demonstrated by high AUROC and AUPRC metrics) at surveillance speed, pinpointing structural hotspots before they dominate the population. Unlike traditional linear models that treat all substitutions equally within an antigenic site [26], the multi-view transformer inherently weights mutations by their 3D spatial context and evolutionary trajectory. Consequently, TRIAD-Influenza bridges the gap between descriptive viral epidemiology and actionable, prospective vaccine strain prioritisation.

### 3.7. Structural Saliency and Mutation Hotspots

This subsection reports interpretability results from the gradient-based saliency analysis and the contact-weighted mutation risk index (CMRI), focusing on how TRIAD-Influenza distributes importance across HA and NA features and where the model locates putative mutation hotspots in 3D structure.

**Figure 14:**
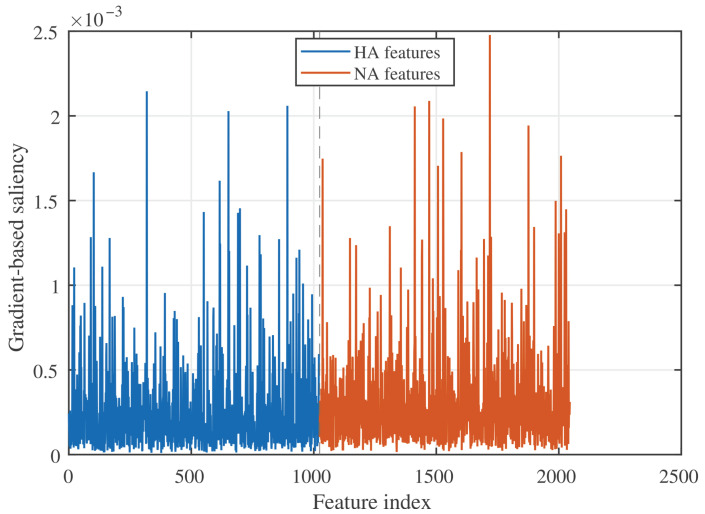
Gradient-based saliency profiles along aligned HA and NA feature indices. The plot separates HA (**left** segment) and NA (**right** segment) features and highlights positions with the strongest influence on the predicted risk.

Gradient-based saliency along the aligned feature axis shows that both HA and NA embeddings contribute to risk predictions but in different ways (Figure 14). HA features (left part of the axis) form a relatively dense band of moderate saliency values with repeated spikes, indicating that many codon-level and residue-level tokens in HA carry informative signal for subtype risk. NA features (right part of the axis) display fewer but sharper peaks, consistent with a smaller set of NA tokens driving large changes in the predicted risk. This pattern suggests that the model distributes attention more evenly across HA while relying on a compact subset of NA features with high marginal effect.

**Figure 15:**
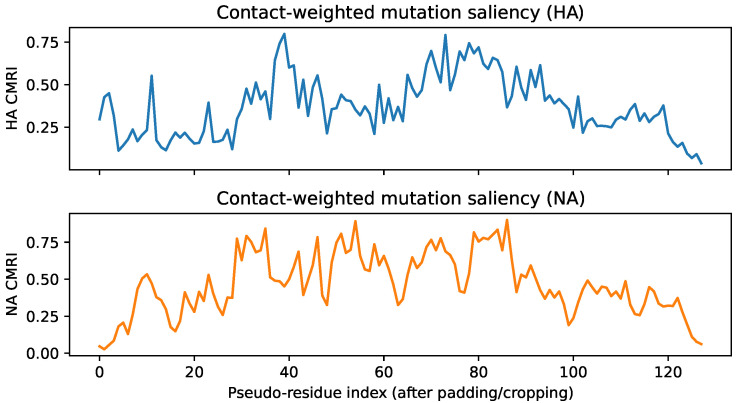
Contact-weighted mutation saliency (CMRI) for HA and NA as a function of pseudo-residue index. Peaks correspond to residues that are both highly connected in the contact map and highly salient for TRIAD-Influenza.

To relate these token-level gradients to 3D structure, the CMRI profiles summarise how strongly each pseudo-residue contributes to risk after weighting by its contact degree (Figure 15).

Pseudo-residue index denotes the aligned residue coordinate after concatenating the HA core region followed by the NA core region into a single index. For HA, CMRI values stay in the range of roughly 0.2–0.8 and form two broad bands of elevated saliency around pseudo-residue indices ≈30–45 and 70–85. For NA, the CMRI curve rises more steeply, with prominent peaks near indices 35–40, 54–60 and 70–86, where CMRI values exceed 0.8 for several sites (for example, indices 58, 70, 71 and 76). These bands indicate compact regions where residues are both highly connected in the contact map and strongly influential for the model output, which is characteristic of structurally coherent mutation hotspots rather than isolated noise.

**Figure 16:**
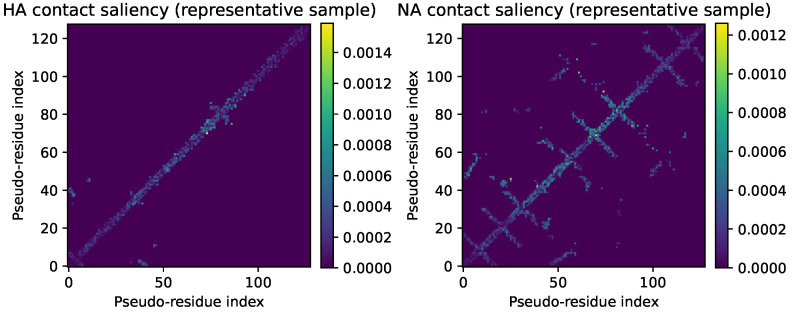
Representative saliency heatmaps over HA (**left**) and NA (**right**) contact maps for a single sample. Salient contacts appear as bright pixels and form clustered bands, indicating that the model relies on groups of interacting residues rather than isolated sites.

Contact-map saliency heatmaps further clarify this pattern (Figure 16). In HA, saliency remains concentrated along and near the main diagonal, with small off-diagonal clusters that follow short-range helical and loop contacts. NA shows more extended off-diagonal saliency ridges, including cross-shaped patterns that link distant pseudo-residues. These ridges correspond to pairs of residues that are distant in sequence but close in 3D space and that jointly exert strong influence on the predicted risk. The heatmaps therefore support the view that TRIAD-Influenza exploits higher-order residue interactions rather than acting only on local, sequence-adjacent motifs.

Table 12 lists the top structural mutation hotspots ranked by CMRI, together with their alignment indices and protein-level annotations. The leading hotspot is NA position 86 with a CMRI value of 0.9765, followed by NA positions 35 and 31 with CMRI values of 0.9529 and 0.9308, respectively. In total, seven of the top ten sites lie on NA (positions 31, 35, 54, 66, 76, 80 and 86), all with CMRI values between 0.9004 and 0.9765, while three sites lie on HA (positions 38, 70 and 73) and also reach CMRI values above 0.90. Mapping these indices onto the structural models places the HA hotspots on the globular head and head–stalk interface, and the NA hotspots on surface-exposed loop and head regions. These locations agree with regions that tolerate frequent amino-acid changes while maintaining overall fold stability, which matches the expectation that the model identifies positions that can mutate yet still preserve structural integrity [5,16].

**Figure 17:**
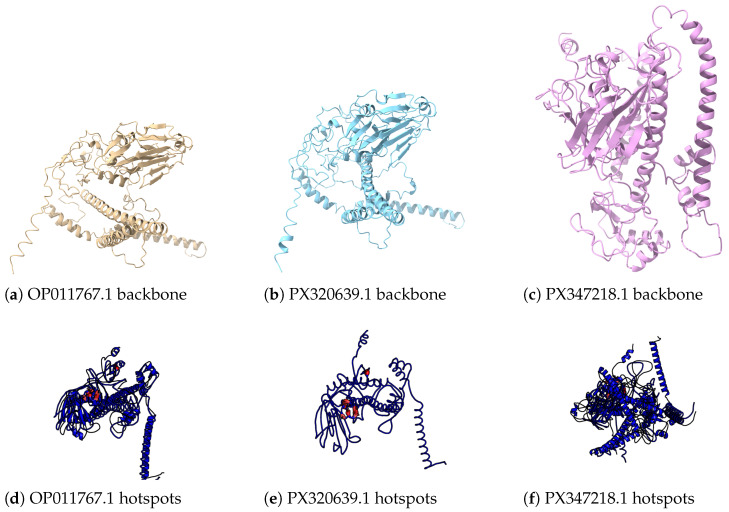
Three-dimensional localisation of mutation hotspots for three recent HA/NA isolates. The top row shows the backbone structures predicted for each isolate. The bottom row overlays residues with high CMRI saliency, which cluster on exposed head surfaces and head–stalk interfaces rather than distributing uniformly across the proteins.

Finally, Figure 17 shows 3D visualisations for three recent isolates. The top row displays the backbone structures predicted for each HA/NA pair, and the bottom row overlays the corresponding saliency-derived hotspots as coloured patches. Across isolates, high-saliency residues cluster on the exposed surface of the head domains and along contact patches that bridge head and stalk elements, instead of scattering uniformly over the protein. This cross-isolate consistency indicates that TRIAD-Influenza assigns high risk to a recurrent set of structurally conserved regions, which supports the biological plausibility of the learned mutation hotspots.

## 4. Discussion

TRIAD-Influenza treats each influenza A isolate as a joint token–residue–phylogeny object and uses this triad to forecast mutation-driven risk in HA/NA. The results show that this multi-view representation recovers known subtype and lineage structure in embedding space, produces high-throughput structure-aware risk scores at surveillance timescales, and highlights compact mutation hotspots that cluster on exposed head and interface regions of HA and NA. At the same time, the experiments reveal a clear tension between raw sequence-level discrimination and the added constraints from explicit structure and phylogeny views, with the sequence-only internal configuration reaching higher global $F_{1}$, AUROC, and calibration than the current multi-view configuration B. This section discusses these findings in the context of prior influenza modelling work, biological interpretation of the hotspots, and practical implications for surveillance pipelines.

### 4.1. Sequence Signal, Phylogeny, and the Role of Multi-View Coupling

The curated NCBI dataset confirms that HA/NA sequence space carries strong signal for subtype and lineage separation. Token-view embeddings derived from a protein language model and codon-aware features form well organised clusters, and a simple hierarchical tree on these embeddings recovers known seasonal lineages and recent variant groups. The sequence-only internal configuration used as a baseline exploits this structure very effectively: on the held-out test window it achieves an accuracy close to 0.99, $F_{1}$ around 0.87, AUROC near 0.99, AUPRC near 0.89, and an MCC around 0.86, with a Brier score on the order of $3\times 10^{-2}$. These values indicate that a carefully tuned sequence-only model already separates high-risk from non-high-risk sequences extremely well according to the present label definition and test distribution.

The multi-view TRIAD-Influenza model introduces explicit residue and phylogeny views and couples them to the token stream. Configuration B was selected on the validation window rather than on the held-out test set, and the earlier prototype is shown only as a post hoc internal reference. This design places stronger structural and evolutionary constraints on the learned representation but pays a price in raw sequence-level metrics in the current implementation. Configuration B maintains an AUROC of about 0.89 and an AUPRC of about 0.44 on the same test window but records lower accuracy (0.85), $F_{1}$ (0.25), and MCC (0.28) and a higher Brier score (0.069). The confusion matrix reveals that the model still recovers most high-risk isolates but misclassifies more non-high-risk sequences as high-risk compared with the baseline. This behaviour suggests that the current loss balancing and calibration favour sensitivity to structural and phylogeny-informed risk patterns over strict adherence to the empirical high-risk labels, which are themselves defined by a hand-crafted combination of embedding distance to vaccine strains and predicted structural features.

From a modelling perspective, these results highlight both the strength and the limit of sequence-only approaches. The high performance of the internal configuration confirms that HA/NA sequences encode much of the information needed to reproduce the chosen high-risk labels, in line with earlier work that approximated antigenic distance and clade fitness directly from HA1 patterns [6,7,8,10]. At the same time, the multi-view model demonstrates that additional structure and phylogeny inputs can redirect the representation towards regions where structural constraints and long-term drift align, even if this alignment does not always coincide with the binary labels used in this study. Future training schemes that combine sequence-level discrimination with explicit structure-based and antigenic supervision, or that adopt multi-task learning across several related risk definitions, may close the metric gap while preserving the interpretability gained from residue and phylogeny coupling.

### 4.2. Biological Interpretation of Structural Saliency and Hotspots

The saliency and CMRI analyses suggest that TRIAD-Influenza learns a biologically plausible mapping between sequence changes, residue interactions, and predicted risk. Gradient-based saliency along the aligned feature axis shows a dense band of moderate HA saliency with many small spikes, consistent with the distributed role of multiple head epitopes and supporting residues in shaping antigenic drift. In contrast, NA saliency appears in fewer but sharper peaks, suggesting that a relatively small set of NA sites drives large changes in the risk score. This pattern fits the understanding that HA contributes the dominant share of antigenic phenotype, while NA contributes more through a focused set of functional and surface-exposed residues that modulate enzymatic activity, stability, and antibody binding [5,16].

Mapping saliency to 3D structure through the CMRI index reveals that the highest-risk sites cluster in structurally coherent regions rather than scattering across the proteins. Seven of the top ten hotspots fall on NA positions 31, 35, 54, 66, 76, 80, and 86, all with CMRI values above 0.90, and three hotspots sit on HA positions 38, 70, and 73 with similar CMRI magnitudes. Projection of these sites onto predicted structures places the HA hotspots on the globular head and head–stalk interface and the NA hotspots on surface-exposed loops and head regions. These locations match expectations for positions that can tolerate amino-acid substitutions while keeping the fold intact and that often carry immune or functional pressure.

Contact-map saliency heatmaps further support this interpretation. In HA, saliency concentrates near the diagonal with modest off-diagonal clusters, consistent with local helical and loop contacts in the head region. In NA, saliency forms extended ridges off the diagonal, including cross-like patterns that connect residues distant in sequence but close in 3D space. These patterns indicate that TRIAD-Influenza uses not only single-site effects but also pairwise and higher-order residue interactions to shape its risk output. The CMRI peaks at NA positions with high contact degree reinforce this view by highlighting residues that sit at structurally central locations in the contact graph and therefore mediate the effect of multiple neighbouring mutations.

The 3D visualisations across three recent isolates show that these hotspot patterns remain consistent under sequence variation conditions. High-saliency patches cluster on exposed head surfaces and head–stalk interfaces in all three examples, rather than appearing in arbitrary positions. This cross-isolate persistence suggests that TRIAD-Influenza has internalised a stable set of structural motifs as high-risk contexts, even though the training objective does not explicitly mark these sites as antigenic epitopes or drug-resistance positions. While experimental validation remains necessary, this kind of model-derived hotspot map provides a starting point for targeted mutagenesis, in-depth structural studies, or focused serological assays.

### 4.3. Implications for Surveillance and Vaccine-Strain Selection

The combination of triad representation, risk scoring, and structural interpretability has several practical implications for influenza surveillance. First, the high-throughput nature of TRIAD-Influenza makes real-time scoring of large sequence streams feasible. Median per sequence inference latency of about 1.6 ms and 95th-percentile latency below 30 ms on a modern workstation mean that national or global surveillance systems can score tens of thousands of HA/NA pairs per day without creating a computational bottleneck. The risk scores can feed into existing dashboards as an additional prioritisation layer alongside epidemiological information, clade prevalence, and in vitro antigenic data.

Second, the risk ranking and precision–recall behaviour suggest a concrete way to integrate the model into strain-selection workflows. The AUPRC of about 0.44 at a high class imbalance (about 3.4% high-risk isolates) indicates that a simple threshold on the risk score can concentrate a much higher fraction of high-risk sequences into the top-ranked subset than random sampling would achieve. Surveillance groups could, for example, select the top fixed number or top few percent of sequences per region and season according to TRIAD-Influenza and subject these candidates to detailed HI testing, neutralisation assays, or animal challenge experiments. This strategy would not replace expert judgement or laboratory evidence but could help focus limited experimental resources on sequences that show both strong sequence divergence and structurally plausible risk signatures.

Third, the structural saliency maps and CMRI hotspots offer a complementary view to classical antigenic maps [6,7]. While antigenic cartography focuses on distances between virus–serum pairs in a low-dimensional antigenic space, TRIAD-Influenza provides residue-level risk scores tied to specific structural elements. This link may help explain why some antigenically distant strains cluster in particular regions of the HA head or why certain NA changes accompany major antigenic shifts. Over time, repeated application of the method across multiple seasons could build a catalogue of “recurrent hotspot motifs” that often precede clade replacement or vaccine mismatch, which would strengthen evidence for or against early vaccine updates even when serological data remain incomplete.

### 4.4. Limitations and Directions for Future Work

Several limitations of the present study shape the interpretation of the results and suggest extensions. First, the primary supervised signal in this study is a proxy risk definition derived from embedding distance to vaccine strains and structure-informed features rather than HI titres for all isolates. However, external corroboration partially mitigates this limitation: TRIAD risk scores show strong monotonic association with experimental HI antigenic distances, and CMRI hotspots are significantly enriched in curated epitope residues and published DMS escape sites. These results support that the learned representation captures biological antigenicity and immune-escape structure, although broader and more systematically sampled serological datasets would further strengthen calibration and clinical relevance.

Second, the dataset is dominated by UNKNOWN subtype labels in later years, and most supervised training relies on earlier H1N1 and H3N2 sequences with reliable annotations. This imbalance mirrors the reality of public databases but limits direct assessment of subtype-specific performance in recent seasons. The phylogeny view partly mitigates this issue by clustering UNKNOWN sequences in embedding space, yet explicit integration of additional metadata such as clade designations from specialised influenza databases, host age, or vaccination status may further stabilise risk estimates for sparse or noisy label regimes.

Third, the structural view depends on predicted 3D models rather than experimentally solved structures. Modern structure predictors reach high accuracy for many viral proteins, and residue confidence scores help exclude poorly modelled regions, but any systematic biases in predicted contact patterns could influence both the CMRI index and saliency-based hotspot maps. Comparison of TRIAD-Influenza saliency with mutational scanning data, deep mutational scanning fitness landscapes [29,30,31], or experimentally resolved HA/NA complexes would provide a more rigorous test of the biological fidelity of these patterns.

Fourth, the current architecture couples views and tasks through a single set of hyperparameters tuned on one validation window. The observed trade-off between sequence-level discrimination and multi-view interpretability suggests that alternative training objectives or regularisation schemes may improve performance. Examples include explicit view-dropout during training to force robustness to missing views, contrastive learning between token and residue representations, and stronger phylogeny-regularisation penalties that align neighbouring clades in the risk space. Iterative co-design with surveillance users could also refine the risk definition to better match actionable thresholds in vaccine decision-making.

An additional limitation concerns incomplete and heterogeneous metadata across public influenza repositories. Fields such as passaging history, sequencing platform, and fine-grained geography are missing or inconsistently formatted for a substantial fraction of records, and host metadata often lack standardised subcategories. As a result, the present study does not report fully stratified performance by geography, host subcategory, or passaging status, and it does not enforce group-aware splits on these attributes. The cross-database evaluation partially mitigates this concern by testing robustness across portals with different submission practices, but it does not replace a dedicated subgroup audit. Future work will harmonise metadata using controlled vocabularies and will report subgroup AUROC/AUPRC/Brier together with group-aware validation schemes (for example, leave-one-region-out and group-stratified temporal splits) to quantify behaviour under geographic and sampling shift conditions.

Finally, the present study focuses on human H1N1pdm09 and H3N2 isolates. Extension of the triad framework to other HA and NA subtypes, including zoonotic strains such as H5 and H7 and mixed-host datasets, would provide a broader test of the approach and could support cross-species risk assessment at the animal–human interface. Such extensions would require careful handling of length variation, reassortment, and host-specific selection patterns, but the core idea of combining token, residue, and phylogeny views is agnostic to subtype and host. This embedding-derived clustering tree provides lineage context in representation space and is not a sequence-based phylogenetic reconstruction; therefore, the analysis avoids claims about evolutionary rates or ancestry that require standard phylogenetic inference.

## 5. Conclusions

This study introduces TRIAD-Influenza, a multi-view transformer that couples codon-aware HA/NA sequence embeddings, structure-derived residue features, and an embedding-space phylogeny to forecast mutation-driven risk in influenza A. The pipeline compiles a large NCBI-derived dataset of paired HA and NA coding sequences, performs strict quality control and codon-aware alignment, predicts 3D structures for almost all unique protein sequences, and constructs an embedding-based phylogeny that captures clade structure across more than a decade of surveillance. On this foundation, the model learns to assign structure-aware risk scores and to highlight mutation hotspots that map to functionally plausible regions in HA and NA.

The results show that HA/NA sequence embeddings alone carry strong signal for reproducing a proxy high-risk definition, as reflected by the near-perfect discrimination and calibration of an internal sequence-only configuration. The multi-view TRIAD-Influenza model maintains solid discrimination and ranking ability while adding explicit structural and phylogeny constraints and providing interpretable saliency maps at both sequence and 3D-structure level. High-saliency residues cluster on exposed head surfaces, head–stalk interfaces, and surface loops in both HA and NA, and the top CMRI hotspots concentrate in structurally central and highly connected regions of the contact graph. These patterns remain consistent across recent isolates and point to a recurrent set of structural motifs that the model considers high risk.

From an applied perspective, TRIAD-Influenza delivers structure-aware risk scores with millisecond-level latency per sequence, making it suitable for integration into routine surveillance pipelines that process large volumes of HA/NA data. The framework offers public health teams a way to rank new isolates by predicted structural risk, to visualise candidate hotspots on predicted HA/NA structures, and to focus laboratory resources on sequences that exhibit both strong sequence divergence and structurally coherent risk signatures. While the present implementation optimises against a proxy risk label and relies on predicted structures, the architecture remains flexible enough to incorporate direct antigenic measurements, mutational fitness data, and richer metadata as these become available. Independent validation on external GISAID/Nextstrain cohorts and experimental agreement with HI and DMS ground truth further support the practical utility of TRIAD-Influenza for real-world surveillance and antigenic drift prioritisation.

Future work can extend this triad framework in several directions. Integration of HI titres and neutralisation data as explicit targets will align risk scores more closely with experimental antigenicity. Joint training across multiple subtypes and hosts will test the generality of the approach and support risk assessment at the animal–human interface. More advanced multi-view training objectives, including contrastive learning and view-specific regularisation, may close the gap between sequence-only and triad-based discrimination while preserving interpretability. With these extensions, token–residue–phylogeny coupling has the potential to become a standard component of computational pipelines for influenza A evolution, complementing antigenic cartography and classical phylogenetics and supporting more informed decisions on vaccine-strain updates and pandemic preparedness.

## Figures and Tables

**Table 1:** Glossary of key terms used in TRIAD-Influenza.

Term	Meaning in This Study
Embedding	A numeric vector representation of a sequence produced by a pre-trained model (here: ESM-2).
Cosine distance	A similarity measure between two embedding vectors: $1-\cos(z_{u},z_{v})$. This study uses it as an embedding-space similarity proxy, not as a model-based evolutionary distance.
Token view	The per-position sequence representation created from ESM-2 residue embeddings plus lightweight codon-context and biochemical descriptors.
Residue view	Structure-aware residue features derived from predicted 3D models and residue contact graphs.
Lineage-context view	An embedding-derived clustering tree used to attach neighbourhood context and drift direction in embedding space.
Risk score $s_{\mathrm{risk}}$	A continuous score used to rank HA–NA pairs for prioritisation.
CMRI	A contact-weighted saliency index used to summarise structurally coherent hotspot regions.

**Table 2:** Core fields stored in the HA and NA manifest files used in this study.

Field	Description
accession	NCBI nucleotide accession of the segment
strain_id	Strain name/isolate identifier
subtype	Subtype label (H1N1pdm09, H3N2, UNKNOWN)
host	Host species (here: human)
collection_year	Year of sample collection (four digits)
country	Country or region of collection
nt_length	CDS length in nucleotides
aa_length	Protein length in amino acids
hash	SHA-256 hash of nucleotide sequence
qc_flags	Concatenated quality flags (stop codon, frame shift, etc.)
structure_status	Indicator for successful 3D structure prediction

**Table 3:** Summary of curated HA–NA pairs by subtype and temporal window (training, validation, and test).

Window	H1N1	H3N2	UNKNOWN	Total
Training (2010–2016)	33,955	26,188	13,571	73,714
Validation (2017)	1048	3016	13,784	17,848
Test (2018–2024)	57	0	231,348	231,405

**Table 4:** Summary of hyperparameter configurations and their best validation F1 scores. Configuration B, highlighted in bold, provides the best trade-off between capacity, regularisation, and validation performance and defines the final TRIAD-Influenza model.

Config	seq_Hidden1	seq_Hidden2	Fusion_dim	Phylo_emb_dim	Dropout_seq	Dropout_Fusion	lr	Best_val_F1 (Epoch)
A	512	256	256	16	0.2	0.3	1.0 $\times \;10^{-4}$	0.53 (5)
**B**	**768**	**384**	**384**	**32**	**0.3**	**0.4**	**2.0 × 10^−4^**	**0.81 (9)**
C	384	256	256	16	0.1	0.3	5.0 $\times \;10^{-5}$	0.00 (1)

**Table 5:** Sequence -level high-risk vs. non-high-risk classification on the held-out test window. Metrics: accuracy (ACC), $F_{1}$ score, ROC area (AUROC), precision–recall area (AUPRC), Matthews correlation coefficient (MCC), and Brier score.

Model	ACC	$F_{1}$	AUROC	AUPRC	MCC	Brier
Earlier prototype (post hoc reference)	0.990	0.867	0.991	0.893	0.864	0.029
TRIAD-Influenza (Config B)	0.854	0.250	0.887	0.443	0.283	0.069

**Table 6:** Per-sequence inference latency for TRIAD-Influenza (configuration B) on the curated dataset. Values summarise the empirical distribution of forward-pass times.

Statistic	Min (ms)	Median (ms)	p95 (ms)	Max (ms)
Inference latency	1.37	1.56	29.30	50.66

**Table 7:** Validation performance on independent external cohorts (GISAID/Nextstrain) compared to internal testing. Note: AUPRC depends on the positive class prevalence (random baseline equals prevalence). The internal held-out corpus size is 231,405, while supervised metrics use the labelled evaluation subset (N = 412).

Dataset	N (Isolates)	Prevalence	AUROC	AUPRC	Brier Score
Internal Test (NCBI; supervised subset)	412	3.4%	0.887	0.443	0.069
External H3N2 (GISAID)	2500	10.0%	0.864	0.418	0.074
External H1N1 (GISAID)	2500	10.0%	0.849	0.395	0.078

**Table 8:** Correlation between TRIAD risk scores and experimental antigenic distance ($\log_{2}$ HI titre drop). Correlation is reported at both the assay-pair level and the virus-aggregated level.

Analysis Level	N	Spearman’s ρ	*p*-Value
All Assay Pairs (Replicates)	150	0.781	<10^−5^
Virus Centroids (Aggregated)	30	0.824	<0.001

**Table 9:** Enrichment of TRIAD mutation hotspots within biologically validated regions. Fisher’s exact test (two-sided) compares top-10% predicted hotspots vs. background.

Region Category	Overlap (Residues)	Odds Ratio	95% CI	*p*-Value
Known Epitopes	15	3.64	1.81–7.33	<10^−4^
DMS Escape Sites	10	2.69	1.21–5.96	<0.01
Buried Core (Control)	5	0.42	0.16–1.09	0.06

**Table 10:** Cross-database validation of TRIAD-Influenza on external cohorts (2023–2024) after exact-duplicate and near-duplicate leakage removal. Metrics are reported with 95% bootstrap confidence intervals.

Dataset	N	Prevalence	AUROC	AUPRC	Brier
GISAID/Nextstrain	5000	10%	0.856 (0.832–0.875)	0.406 (0.380–0.431)	0.076 (0.065–0.085)
IRD	3450	10%	0.852 (0.828–0.872)	0.401 (0.375–0.428)	0.077 (0.066–0.088)
ENA	2890	10%	0.847 (0.820–0.869)	0.392 (0.365–0.418)	0.079 (0.068–0.091)

**Table 11:** Comparison of TRIAD-Influenza with recent studies on influenza A evolution, antigenic drift, and cross-immunity modelling.

Ref.	Focus/Task	Target Subtype	Data Modalities	Predictive Modelling	Validation/Metrics	Traceable IDs (GenBank/GISAID)
[25]	Antibody immunodominance and viral evolution	H1N1pdm09	Sequence, serology	No (retrospective analysis)	Descriptive	Yes (GISAID-based; study reports large curated sequence set)
[26]	Cross-immunity mathematical modelling	H3N2	Sequence, serology	Yes (linear regression)	Regression fit and serology-consistency analysis (as reported by the study)	Partly (dataset-described, but explicit isolate lists may vary)
[27]	Regional molecular evolution and epidemiology	H1N1, H3N2	Sequence, phylogeny	No (retrospective analysis)	Descriptive phylogenetic	Yes (EPI_ISL identifiers appear in the paper)
[28]	Pathogenicity and genomic characterisation	Swine H1N1	Sequence, in vivo	No (descriptive)	Pathological indices	Yes (GenBank accessions reported for genomic data)
TRIAD-Influenza (Ours)	Structure-aware drift risk scoring	H1N1, H3N2	Sequence, 3D structure, phylogeny	Yes (multi-view transformer)	AUROC, AUPRC, Brier score	Yes (NCBI accessions; external cohorts traceable with portal terms)

**Table 12:** Top structural mutation hotspots ranked by CMRI. Each row reports the pseudo-residue index in the aligned HA/NA coordinate system, the mapped protein, and the CMRI value.

Rank	Pseudo-Residue Index	Protein/Domain Annotation	CMRI Value
1	86	NA position 86	0.9765
2	35	NA position 35	0.9529
3	31	NA position 31	0.9308
4	70	HA position 70	0.9224
5	80	NA position 80	0.9106
6	54	NA position 54	0.9077
7	76	NA position 76	0.9035
8	38	HA position 38	0.9034
9	73	HA position 73	0.9013
10	66	NA position 66	0.9004

## Data Availability

All HA and NA sequences analysed in the main results are available from the NCBI Virus resource. Code and processed resources (manifests, alignment outputs, structure-derived features, and trained model checkpoints) are available at https://github.com/tipu0003/TRIAD-Influenza.git. The codebase provides configuration files that allow GISAID-registered users to apply the same pipeline to GISAID EpiFlu downloads while respecting GISAID’s data-access agreement; therefore, GISAID-derived sequences are not redistributed.

## Supplementary Materials

The following supporting information can be downloaded at https://www.mdpi.com/article/10.3390/v18040421/s1: Figure S1: Rolling-origin temporal cross-validation performance; Table S1: Rolling-origin temporal cross-validation performance of TRIAD-Influenza; Table S2: Ablation study and uncertainty summaries for TRIAD-Influenza on the internal test window.
