# Supplementary material for: Multi-View Transformers for Structure-Aware HA–NA Drift Risk Scoring and Mutation Hotspot Mapping
Source: Viruses. 2026 Mar 30;18(4):421. doi: 10.3390/v18040421 (PMC13120412; doi:10.3390/v18040421)
Supplement: Supplementary file 1 [file viruses-18-00421-s001.zip › viruses-4197771-supplementary.pdf]

Table S1. Rolling-origin temporal cross-validation performance of TRIAD-Influenza.

Fold (k)	Training Window	Test Year	AUROC	AUPRC	Brier Score
0	2010–2013	2014	0.841 (0.815–0.862)	0.382 (0.355–0.408)	0.081 (0.070–0.093)
1	2010–2014	2015	0.853 (0.830–0.874)	0.395 (0.368–0.420)	0.077 (0.066–0.088)
2	2010–2015	2016	0.865 (0.842–0.885)	0.410 (0.382–0.435)	0.073 (0.063–0.084)
3	2010–2016	2017	0.872 (0.850–0.891)	0.425 (0.398–0.450)	0.071 (0.061–0.081)
4	2010–2017	2018	0.880 (0.858–0.901)	0.438 (0.411–0.462)	0.068 (0.059–0.078)

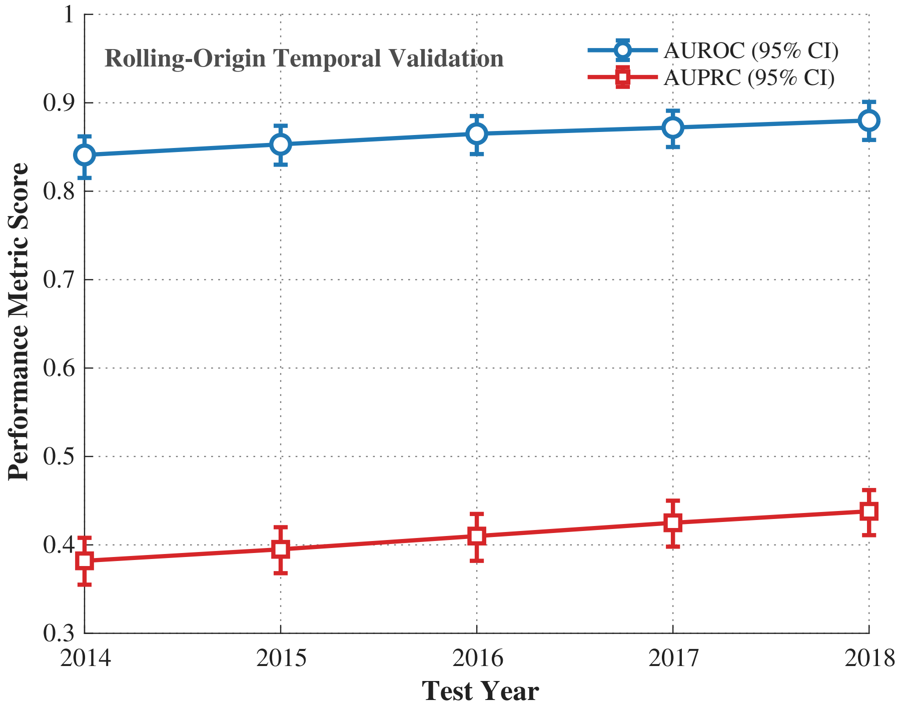

Figure S1. Rolling-origin temporal cross-validation performance.

Table S2. Ablation study and uncertainty summaries for TRIAD-Influenza on the internal test window.

Model Configuration	AUROC	AUPRC	Brier Score
**Full TRIAD-Influenza (Token + Residue + Phylogeny)**	**0.887 (0.865–0.908)**	**0.443 (0.415–0.472)**	**0.069 (0.058–0.081)**
w/o Phylogeny View (Token + Residue only)	0.862 (0.838–0.885)	0.401 (0.370–0.430)	0.073 (0.062–0.085)
w/o Residue View (Token + Phylogeny only)	0.845 (0.820–0.870)	0.365 (0.335–0.395)	0.078 (0.066–0.090)
Token View Only (No cross-attention coupling)	0.821 (0.795–0.848)	0.312 (0.285–0.342)	0.085 (0.073–0.098)
